# Stigma Mutation: Tracking Lineage, Variation and Strength in Emerging COVID-19 Stigma

**DOI:** 10.1177/13607804211031580

**Published:** 2021-08-24

**Authors:** Hannah Farrimond

**Affiliations:** University of Exeter, UK

**Keywords:** COVID-19, prejudice, public health, stigma, process, stereotypes, anti-Asian, China, discrimination

## Abstract

In this article, I propose a novel theoretical framework for conceptualizing pandemic stigma using the metaphor of ‘mutation’. This metaphor highlights that stigma is not a static or fixed state but is enacted through processes of continuity and change. The following three orienting concepts are identified: (a) lineage (i.e. origin narratives and initial manifestations are created in relation to existing stigmas, stereotypes, and outgroups), (b) variation (i.e. stigma changes over time in response to new content and contexts), and (c) strength (i.e. stigma can be amplified or weakened through counter- or de-stigmatizing forces). I go on to use this metaphor to offer an analysis of the emergence of COVID-19 stigma. The lineage of COVID-19 stigma includes a long history of contagious disease, resonant with fears of contamination and death. Origin narratives have stigmatized Asian/Chinese groups as virus carriers, leading to socio-political manifestations of discrimination. Newer ‘risky’ groups have emerged in relation to old age, race and ethnicity, poverty, and weight, whose designation as ‘vulnerable’ simultaneously identifies them as victims in need of protection but also as a risk to the social body. Counter-stigmatizing trends are also visible. Public disclosure of having COVID-19 by high-status individuals such as the actor Tom Hanks has, in some instances, converted ‘testing positive’ into shared rather than shamed behaviour in the West. As discourses concerning risk, controllability, and blame unfold, so COVID-19 stigma will further mutate. In conclusion, the metaphor of mutation, and its three concepts of lineage, variation, and strength, offers a vocabulary through which to articulate emergent and ongoing stigma processes. Furthermore, the concept of stigma mutation identifies a clear role for social scientists and public health in terms of process engagement; to disrupt stigma, remaking it in less deadly forms or even to prevent its emergence altogether.

‘A major outbreak of novel, fatal epidemic disease can quickly be followed . . . by plagues of fear, panic, suspicion and stigma’. (Strong, 1990: 249)

Pandemics and stigma go hand in hand. It is not surprising, therefore, that the spectre of stigmatized health-care workers or sufferers, familiar from prior epidemics, is returning. However, celebrities and politicians’ openness about their ‘Covid status’ is striking. ‘Hanx’ (Tom Hanks) and his friends do not appear afraid of gaining a ‘spoiled social identity’ (Goffman, 1963) as a result of being COVID-19 positive. Stigma is endemic alongside new contagious disease. The sudden emergence of a new threat throws social life into disarray. The chaos also makes tracking and analysing stigma difficult. In this article, I outline a novel theoretical framework for analysing the emergence of pandemic stigma using the metaphor of ‘mutation’. This metaphor draws attention to (a) stigma lineage, its emergence in relation to prior stigmas and origin stories; (b) stigma variance, its change over time, with cultural and temporal variation; and (c) stigma strength, its amplification, or weakening through de- and counter-stigmatization. I then use this theory to offer an analysis of the initial unfolding of COVID-19 stigma as it has emerged within complex, social media driven, globalized local worlds, highlighting the opportunities for intervention.

## Stigma mutation: theory

‘Man’s yesterday may ne’er be like his morrow; Nought may endure but Mutability’. (Shelley, 1885)

### Pandemic stigma literature

In this section, I review current thinking about the sociology of stigma, particularly the recent emphasis on the ideological and institutional creation of stigmatized identities beyond the interpersonal interactions first articulated by Goffman (e.g. Link and Phelan, 2001; Parker and Aggleton, 2003; Scambler, 2018; Tyler, 2020). Stigma is a state of social devaluation, being designated as ‘lesser’, due to a characteristic, difference, or membership of a group (Goffman, 1963). Stigmatization occurs when others behave differently towards the person, from overt discrimination to micro-aggressions. It is also experienced internally as ‘felt’ stigma, either as self-stigma (feeling shame towards oneself) or perceived stigma (anticipating others stigmatizing views or behaviours; Hammarlund et al., 2018). In other words, stigma consists of possessing a derogated social identity with external and internal consequences; however, these differ widely in how they manifest.

Recent sociological theories have emphasized that the creation of derogated social identities is not just a matter of interpersonal dislike or fear, but is embedded in wider socio-cultural representations of the ‘other’. We need to ask who, culturally and politically, is doing the stigmatizing, and why? For example, Scambler argues recent discourses about the responsibility of the sick and disabled for their own plight is directly linked to neo-liberal values underlying capitalist governance of Austerity Britain (Scambler, 2018). The ‘weaponizing’ of stigma, pairing the shame of state dependency with blame, diverts attention away from other misuses of power. Similarly, Tyler (2020) has drawn attention to the reproductive historical nature of power in racial inequalities. The relevance of this theorization is acute given COVID-19’s acceleration of the ‘othering’ of displaced, migrant, and ethnic minority groups (Roelen et al., 2020). The current focus on the operationalization of power within stigma theory reminds us that stigma emerges from complex socio-historical contexts and power relations; speaking about ‘COVID-19 stigma’ may overly simplify and reify what is occurring, and also hide questions about why.

In relation to disease, stigma has been identified across a large range of conditions; for example, mental illness (Hayward and Bright, 1997), chronic pain (Jackson, 2005), and other non-communicable diseases (Rose et al., 2017). However, the contours of stigma in relation to communicable or infectious diseases, such as viral epidemics, are particularly well-delineated. Orienting around a fear of contamination, an extensive literature has documented the stigma of people with Human Immunodeficiency Virus (HIV)/Acquired Immunodeficiency Syndrome (AIDS) (e.g. Parker and Aggleton, 2003) as well as for other epidemic or pandemic diseases, such as Tuberculosis (TB), Severe Acute Respiratory Syndrome (SARS), Middle East Respiratory Syndrome (MERS) and Ebola (e.g. Mak et al., 2006). For example, the stigma of Ebola resulted in the shunning of survivors, health-care workers, and occasionally, the entire village (Hewlett and Amolat, 2003). Pandemic stigma is highly problematic in disrupting efforts to contain transmission. Being stigmatized causes psychological distress, making groups the target of social rejection and exacerbates inequalities (Parker and Aggleton, 2003). Furthermore, provoking shame interferes with prevention and containment behaviours (Dolezal and Lyons, 2017). Affected individuals may avoid being tested, hide test results, or fail to adopt safer practices. Stigma prevention is consequently a core public health strategy for pandemic management. The COVID-19 virus has created an urgency to identify emergent stigma, given its potential to entrench socio-political inequalities and inhibit virus control.

### The metaphor of mutation

My aim in this article is to articulate the ‘epidemic of stigma’ that Strong has identified, paying particular attention to the way that stigma emerges, mutates, and changes in response to contexts. Although much work on stigma has measured individual and aggregate stigma, some research has started to articulate the social processes underlying pandemic stigma (e.g. Parker and Aggleton, 2003; Roelen et al., 2020). Anthropological work on prior epidemics has also highlighted the importance of cultural context through specific case studies (e.g. Brewis and Wutich, 2019; Hewlett and Amolat, 2003). This article takes a wide lens approach, offering a novel metaphor with which to think through stigma emergence in global and local contexts; and a borrowed (from biological science) vocabulary to articulate its contextual and temporal unfolding. Offering up a theoretical framework that considers stigma widely will allow a focus on particular elements of stigma, or a particular variant in one locality, but with the awareness of the interconnectedness of these enactments in 21st-century life. I suggest three aspects of the mutation metaphor are useful as orienting concepts:

1. Stigma Lineage: Lineage denotes, biologically, the evolutionary heritage of a species; each version has evolved from its predecessor. I suggest that stigmas, like viruses, have lineage. For example, novel epidemic stigma does not develop in isolation, but as part of a suite of already stigmatized epidemic diseases, ranging from older forms of plague (e.g. Black Death, bubonic plague) and more recently HIV/AIDS, Ebola, SARS, and MERS. New knowledge is absorbed into and recreates older representations of disease, a process known in social representation theory as ‘anchoring’ (Jodelet, 2008). Terms such as ‘plague’ reoccur and are remade in relation to novel diseases, thus mutating from the original into something different but related. Not only does stigma have ‘lineage’ from other contagious diseases; stigma is produced in relation to other stigmas. Charting the lineage of novel pandemic stigma, therefore, involves tracking and identifying not only how the past is configured in current representations, but also how the present is reconfigured by them. ‘Origin stories’, which tell us about the origins or causes of a given disease outbreak, also function to thread together elements of past epidemics and prejudices, and entwine these together, continuing the lineage of othering fundamental to stigmatization. Lineage is thus fundamental to understanding new stigma mutation. Stigma’s power in the present is linked to prior manifestations and institutional drivers. Identifying the anchors of stigma helps make sense of its present forms.
2. Stigma Variation: Not only do biological viruses have lineage, they also mutate, sometimes fast, sometimes slow, producing new variants. Stigma is also not static. Its content changes over time, as more information is known, as fears emerge in relation to ‘outgroups’ and as people project these fears onto the ‘Unhealthy Other’ (Crawford, 1994). We also need to consider who are the social actors producing stigma content, and for what ideological, political, or other purposes. Public health actors should not be exempted from scrutiny. Health promotion has often drawn on fearful or disgusting imagery, such as diseased or repulsive bodies, to motivate behaviour change (Lupton, 2015). Variants also occur in different cultural contexts, particularly along what has been termed social ‘fracture-lines’ (Roelen et al., 2020) of localized historical prejudice and inequality. A theory of stigma should be able to reflect the contextual and cultural variation in stigma as well as its global and common forms. Finally, stigma varies over time; as with biology, new variants can emerge slowly or very quickly. Recent work is starting to articulate the importance of temporality for stigma (e.g. Bonnington et al., 2017; Mbonye et al., 2013). Previous research has tended to ‘slice’ data at quite long intervals, sampling stigma over years or decades. A theory of pandemic stigma mutation needs to account for its abrupt emergence, amplified by global media, but also identify stability and longer term variation; in other words, articulate BOTH continuity and change over varying time-scales.
3. Stigma Strength: Stigma mutation into stronger, more virulent forms, is not inevitable. Biological variants can become less, as well as more, harmful over time. Similarly, stigma is often amplified during particular historical moments, such as a pandemic, but can also weaken over time and be mitigated through active anti-stigma interventions or longer term historical shifts. Active interventions include tackling self-stigma towards oneself as a sufferer, engaging with communities, developing non-stigmatizing health-care services, and social change through mass media (Pulerwitz et al., 2010). Moving to consider ‘de-stigmatization’, the reversal of devaluation, which has received less attention in the literature, Clair and colleagues have identified key events and social conditions that underlie a lessening of prejudice towards African Americans in the West over past decades (Clair et al., 2016). However, as the racially driven US industrial prison complex demonstrates, stigma and prejudice are often remade in less overt but no less deadly ways (Handra, 2020). This leaves the potential for sudden amplification (e.g. the death of George Floyd; the emergence of ‘Black Lives Matter’). Two points about stigma strength are important here. One is that in a new pandemic, stigma is not fixed and is highly dynamic and malleable. We need to pay attention to both what is driving stigmatization but also what is actively preventing stigma (e.g. public health interventions) or exerting cultural pressure against it (counter-stigmatizing forces). Second, we also need to pay attention to which social actors (or events) are having a counter or de-stigmatizing effect. Stigma is a form of status loss (Link and Phelan, 2001). Conversely, if high social status individuals possess a devalued attribute, they are often cushioned from stigmatizing effects. They can also create new, more positive, social norms, from a place of socio-economic and cultural capital. A processual account of stigma mutation, therefore, pays attention to complex change over time, including both amplifying and counter or de-stigmatizing forces.

In this section, I have explained why stigma mutation may be a useful conceptual metaphor to consider stigma change over time. However, I have some reservations. Cultural studies have charted the use of the term ‘mutation’, from the 1950s radiation effects to current usage and this has progressively become more negative (Condit et al., 2002). Mutation as it appears in literature also carries a sense of monsters, ‘mutants’ which are neither human, nor anything else. Shelley’s poem was quoted in his wife’s study of transformative horror, Frankenstein. However, stigma IS a negative social phenomenon, not a positive one. Mutation is more recently and neutrally used in biology to describe changes in genes, the effects of which are often negative, but can also be advantageous (e.g. as part of genetic variation or evolutionary adaptation). With respect to viruses, there are already multiple genetic variants of COVID-19. The key reason, therefore, that I have stuck with the metaphor is not just the notion of mutation, but of mutability, the possibility and likelihood of change. The notion of stigma mutation avoids a reductionist sense of inevitability about COVID-19 stigma. It asks, what can we do differently? Where can we engage and disrupt stigma processes? As social scientists better understand the processes of stigma emergence and mutation, the opportunities to prevent stigma forming, embedding, or reoccurring are greater. Furthermore, understanding stigma in terms of lineage, variation, and strength has applicability beyond the immediate pandemic as a way to conceptualize stigma continuity and change. In the next section, I offer a worked through example of the utility of the mutation metaphor, at a particular time point (March 2020 to May 2021) from a Western (UK) perspective, to illustrate its portability to stigma theory in general.

## Analysis of emergence and mutation of COVID-19 stigma

Since my initial draft in summer 2020, there has been a plethora of literature published to indicate COVID-19 stigma emergence. Broadly, this literature is of two types. One is policy-driven work which highlights COVID-19 stigma as a core issue for public health (e.g. Logie and Turan, 2020; Sotgiu and Dobler, 2020; Van Daalen et al., 2021). The other offers early empirical evidence of COVID-19 stigma, for example, documenting hostility towards survivors in Kashmir and Latin America (Bagcchi, 2020; Dar et al., 2020); stigmatizing behaviour towards health-care workers (Dye et al., 2020; McKay et al., 2020) and contamination fears over death practices in Egypt (Abdelhafiz and Alorabi, 2020), Indonesia, and Iran (World Health Organization, 2020). Prejudice towards perceived ‘origin groups’ and social practices such as mask-wearing has also been identified (Ma and Zhan, 2020). Collectively, this work establishes that COVID-19 stigma has begun to emerge and that challenging it is important. However, often COVID-19 stigma is presented as a relatively fixed entity; something that now exists; ‘health-care workers are stigmatized’, even though there is huge variation and lack of universality about these experiences (although, see Bagcchi, 2020; Roelen et al., 2020). What I offer in the next sections, therefore, is a nuanced account of COVID-19 stigma emergence which pays attention to its lineage in past epidemics, and accounts for important culture-specific variance.

## Stigma lineage

### Lineage of contagious diseases

COVID-19 is part of a lineage of highly feared contagious diseases: Ebola, SARS, MERS and HIV/AIDS and other viruses such as flu. Underlying this stigma is the ‘threat’ they pose (Jones et al., 1984). That said, although COVID-19’s mortality rate is considerable (current estimates can be found at: ArcGIS.com/apps/dashboard, accessed 04 June 2021), the individual risk of death for sufferers is not as high as in other epidemics such as Ebola (e.g. with 50% of those infected dying), nor the toll currently as high as for HIV/AIDS. However, COVID-19, in all of its variants, is relatively contagious compared with SARS or MERS (Sanche et al., 2020). Contagiousness is an important dimension of disease stigma, as it threatens others, not just the self (Jones et al., 1984), invoking symbolic as well as physical fears of pollution (Douglas, 1966). In terms of threat and contagion, therefore, COVID-19 has strong lineage with other epidemics.

Another core dimension of disease stigma is controllability; the extent to which the condition is deemed the bearer’s responsibility (Joffe and Staerkle, 2007). Attributions of controllability vary, for example, HIV/AIDS is deemed more controllable than SARS or TB (e.g. Mak et al., 2006). However, the construct of controllability replicates existing societal biases. One of the primary methods for controlling COVID-19 is social distancing, through staying at home or shielding. However, only more advantaged workers with permanent jobs can work from home, leaving those with more precarious public facing jobs, such as poorer women and ethnic minority groups, disadvantaged (Fouad et al., 2020). Furthermore, attributions of controllability are moral. Blame ensues if people do not follow what is considered sensible and reasonable. Tom Hanks was not condemned for spreading COVID-19 to a new population in Australia in March 2020. However, by February 2021, celebrity apologies for breaking lockdown rules were ubiquitous (e.g. British celebrity Rita Ora, https://www.bbc.co.uk/news/entertainment-arts-55213784, accessed 27 February 2021). Perceptions of controllability, and thus of culpability, have mutated over time. Those who transgress ‘reasonable avoidance’ are blamed, especially if their risk factors are also perceived to be controllable or ‘achieved’ (Falk, 2001), such as obesity (Flint, 2020).

Although similar, COVID-19 stigma does not share complete lineage with other contagious diseases. HIV/AIDS has been multiply stigmatized through its association with perceived deviant sexual behaviour in outgroups (the so-called ‘gay plague’) and, underlying this, connotations of biblical punishment (Crawford, 1994). COVID-19 is not classified as a sexually transmitted disease; its disclosure correspondingly less taboo. However, discourses of ‘defying nature’ have been remade for COVID-19 times, for example, apocryphal (and untrue) tales abounded of dolphins swimming in the canals of Venice. Pollution levels dropped as capitalist production was locked down, at least temporarily. The discourse of plagues as acts of ‘purification’ of a sinful world continues.

Second, the rhetorical use of lineage for socio-political purposes has been pronounced. Some diseases such as (the Big C) cancer are metaphorically more scary, beyond biology; others such as flu less so (Sontag, 1988). Early on in the pandemic, Donald Trump, then President of the US, compared COVID-19 to the flu, a largely non-stigmatized and tolerated illness, minimizing the sense of risk. However, by mid-April 2020, Trump’s language had shifted towards far more feared associations: COVID-19 was ‘a great and powerful plague’ which had come from outside, in this instance, a ‘Chinese plague’. Leaving aside the xenophobia of this language (which I consider later), this anchors COVID-19 into a long line of ‘plagues’, and with them, connotations of cataclysm, chaos, and the end of the world.

The anchoring of new knowledge into older representations is as much an emotional process as a rational one. COVID-19 is not always visible; asymptomatic transmission is common. However, it pulls strongly on the underlying emotions of stigma: fear and disgust (Lupton, 2015). It reminds us of bodily processes, what Rozin has called ‘animal-reminder’ disgust (Rozin et al., 2008). Transmission occurs through breath and contact with others; dying of COVID-19 has been described as ‘drowning’. Disgust towards being polluted through breath, which fails to observe bodily boundaries, is noted in other stigmas, such as smoking (Farrimond and Joffe, 2006). COVID-19’s invisible transmission, which can leave no ‘mark’ on the carrier, provokes chaotic fear of contamination. Identifying the origins of the disease offers an opportunity to make order out of chaos but also to assign blame, particularly towards those perceived to have started it, such as ‘Patient Zero’ and original carrier communities.

### Lineage in origin narratives

The identification of the ‘other’ as the primary source of risk is pronounced within origin narratives. Origin (or outbreak) narratives are cultural tropes, amplified in film and media, which explain the emergence of novel disease, track its contagious progress, and end (ideally) with its containment by scientists and epidemiologists (Wald, 2008). Origin stories often perpetuate the lineage of already existing stigma. They are moral stories. Accusations of immorality and blame are projected across entire groups, away from the self (Crawford, 1994). Higher status groups also use outbreak narratives to shore up their own power; for example, the Global North tends to downplay the social determinants of disease and blame it instead on the anti-modernity of the Global South (Wald, 2008).

The origin narratives of COVID-19 in the West show rapid ‘othering’; identifying the source of COVID-19 as Chinese, and triggering a wave of anti-Chinese and Asian sentiment towards them as ‘carrier groups’ (e.g. Darling-Hammond et al., 2020; Ma and Zhan, 2020; Van Daalen et al., 2021; Wu et al., 2021). The first, most potent COVID-19 origin story is that the virus mutated from animals to humans in Wuhan, China, through a ‘wet market’ which kills and sells animals for consumption. The US rock singer Bryan Adams had to apologize after a rant against ‘bat eating, wet market animal selling, virus making greedy bastards’ (Beaumont-Thomas, 2020). The wet market story has all the elements of potential stigmatization from a Western perspective: threat, foreign ‘other’, dark, and disgusting practices relating to animals. It also fits with Weal’s hypothesis that the Global North shores up its own cultural ideologies through identifying others as anti-modern. The idea that Asia is a source of plague-type viruses already has lineage, for example, in terms such as ‘Asian flu’. COVID-19 has thus triggered existing racial prejudices towards Asians, increasing the mental health burden on Chinese/Asian people (Wu et al., 2021).

The ideological ‘weaponising’ (to use Scambler’s term) of anti-Asian stigma for political gain is also visible. Conservative elites in the US ‘racialized’ the pandemic, with then President Trump using phrases such as the ‘Wuhan Virus’, ‘China Virus’, ‘Chinese Plague’, and even ‘Kung Flu’ (Ma and Zhan, 2020; Reny and Barreto, 2020). The World Health Organization (WHO) condemns using location names as stigmatizing, offering a different nomenclature based on Greek letters to denote key variants (https://www.who.int/en/activities/tracking-SARS-CoV-2-variants/, accessed 02 June 2021). The pejorative labelling by Trump was arguably deliberate, part of wider political actions against China, such as withdrawing from the perceived Chinese sympathetic WHO and starting a trade war; it also worked to divert attention from domestic deficiencies in COVID-19 policy. Origin narratives are thus not created from scratch, but from lineages of socio-political outgroup discrimination. For example, in India, existing tensions over Northeast Indians, and their identification as non- or ‘unwanted’ Indians, has been exacerbated by COVID-19 (Haokip, 2021) with a similar patterning emerging for Chinese South Americans in Chile (Chan and Strabucchi, 2021).

The origin story of COVID-19 has already mutated. Theories that began as conspiracies, such as the releasing of the virus from a Wuhan laboratory have been resurrected and disputed by the WHO, US and UK state agencies. More positive discourses which position Asian cultures such as Singapore and South Korea as ‘good at pandemics’ have also emerged in relation to technologically superior track and trace systems and the cultural propensity to wear masks, although this label is a precarious one, given that often apparently successful nations can go on to experience later COVID waves. Political resistance to denigration is also occurring, through anti-stigma campaigns in the US such as ‘Stop Asian Hate’. In the next section, I outline some of the ways COVID-19 stigma is mutating beyond the first wave of origin stories to identify new ‘risky’ groups.

## Stigma variation

### Beyond origin stories: newer ‘risky’ groups

Origin stories emerge quickly and potently in the first wave of any new virus. However, the patterning of the virus, and thus, the patterning of stigma, often changes again as newer ‘carrier groups’ are identified. Again, unsurprisingly, this identification of deviance often follows existing fault-lines (Roelen et al., 2020); this is not co-incidental, but expected to some extent, as bodies which are already disadvantaged are more likely to be adversely affected by viruses.

The association between the virus and patterns of inequality is particular pronounced for COVID-19 (e.g. Paremoer et al., 2021). The risk of severe disease and death is highest for older people (particularly the ‘oldest old’), those with pre-existing health conditions, socio-economically disadvantaged people, Black and ethnic minority groups, those with severe psychiatric conditions, the displaced/homeless and the obese. This list of COVID-19 ‘risky groups’ reads as a list of stigmatized groups within Western society. What, then, are the implications for COVID-19 stigma? One implication is that stereotypes for already stigmatized groups are at hand. New stereotypes, integrating COVID-19, are thus easily created. Second, identifying these groups as ‘at risk’ epidemiologically, is socially ‘risky’ for them. Stigma often takes the form of projections of risk and unhealthiness onto whole communities, not just those affected (Crawford, 1994). These groups then become the repositories of blame for society’s inability to control the virus.

Attributions of blame are particularly acute if membership of the group is deemed controllable, such as obesity (Flint, 2020). Weight stigma is heavily entrenched in Western society, carrying connotations of laziness and loss of control. Two key assumptions underpin the moral evaluation of ‘fatness’; that obesity is a costly threat to national and global health; and that obesity is preventable, hence the moral censure of those who ‘let themselves’ become obese (Throsby, 2007). Even within public health, individualistic discourses of lifestyle change position the obese as blameworthy, downplaying the structural obesogenic environment (Lupton, 2015). Weight stigma is being replicated in stigmatizing media discourses on COVID-19 risk (Flint, 2020). The UK prime minister, Boris Johnson, after recovering from COVID-19 himself as an overweight person, declared a ‘War on Fat’, rhetoric reminiscent of previous, often stigmatizing, anti-obesity campaigns. More recently, high body mass index (BMI) has been included as a qualifier for earlier vaccination, leading to moral debates. It is difficult to argue COVID-19 stigma is driving weight stigma in the West when the latter is already so pronounced. However, COVID-19 represents the further retrenchment of obesity as a prime risk to the social body that Throsby identified. Furthermore, obesity also patterns by social inequalities, creating a ‘constellation’ of COVID-19 stigma for multiply vulnerable groups.

The identification of a given group as being ‘at risk’ or ‘vulnerable’ for COVID-19 creates a tension for already stigmatized communities. On one hand, being identified as ‘at risk’ allows groups to be protected, for example, appearing on ‘shielding’ lists and having vaccination priority. On the other hand, stigmatized groups risk being identified WITH the disease, so that they become symbolic of it. Theirs is a fragile social identity which simultaneously identifies them as victims in need of protection but also as a risk to the social body, potentially leading to devaluation and discrimination. This is why I refer to them as ‘risky’ groups, rather than ‘at risk’ groups, as from a sociological point of view, their status as ‘at risk’ is risky for them, in terms of derogated social identity.

This vulnerability or danger tension is not just relevant to already stigmatized groups. Often considered ‘heroes’ within disaster scenarios, substantive global reports of the stigmatization of COVID-19 health-care workers are emerging (e.g. Dye et al., 2020; McKay et al., 2020; Taylor et al., 2020). In COVID-19 times, doctors and nurses simultaneously tread a path of being both ‘heroes’ but also a contamination risk, risking social disapproval and discrimination if they wear uniforms outside medical settings (Dolezal and Rose, 2020). Dolezal and Rose interpret this through the work of Kearney who suggests that moments of terror or war create simultaneous ‘gods’ and ‘monsters’ out of ‘others’; health-care workers are therefore positioned as both saviours and sinners because of their close contact with the infected.

The portrayal of older people in COVID-19 also draws on the ‘gods/monsters’ dichotomy. Existing stereotypes of elderly people orient around them as fragile, vulnerable but sweet (e.g. ‘doddering but dear’; Cuddy and Fiske, 2002). Alternatively, they can be positioned as heroic, exemplified by the media storm surrounding veteran Sir Tom Moore who completed 100 laps of his garden for his 100th birthday, raising 22 million pounds for the National Health Service (NHS), subsequently dying with COVID. The veneration of Sir Tom fits with a particular version of British history, prominent within Brexit rhetoric (the leaving of the UK from the European Union), of ‘Blitz Spirit’; a pandemic version of wartime propaganda. War metaphors reoccur as useful political narratives in epidemics to promote collective action but also to suppress less palatable narratives such as political unpreparedness (De Waal, 2021). In the story of Sir Tom, wartime rhetoric is also linked to the NHS representing the core of Britishness; itself linked to longer histories of imperialism (Fitzgerald et al., 2020). The heroic can, however, also be stigmatized. Very elderly people cost money. As the economic impact of COVID-19 lockdown policies are counted, these discourses of ‘value’ have become more pronounced, particularly concerning those considered not fully human, such as people with dementia, ‘the living dead’ (Behuniak, 2011). The prioritization of the oldest old for COVID-19 vaccination in the UK can thus be read simultaneously in two ways: as an indicator of the preciousness of the oldest old in our society and as the prioritization of the most expensive health-care group; the two narratives are not mutually exclusive. Old people are thus both the embodiment of the NHS, but also its biggest threat. In the next section, I move beyond mutations visible in the UK and the US (those most obvious from my interpretive lens of UK lockdown writing) to consider further emergent cultural variations in stigma.

### Cultural variation

Paying attention to cultural variation goes beyond considering context in the narrow sense. Rather, the enactment of stigma is understood as a set of embedded and embodied cultural practices. Thus, stigma is localized, pulling on older lineages of division and discrimination. For example, in mid-2020, discourses emerged in many Eastern European countries concerning the risk their diaspora posed in terms of bring in COVID-19 when returning home (Paun, 2020). Such discourses were seized on by political groups to further politicised (or to use Scambler’s term ‘weaponised’) stigma against those who were denigrated for ‘abandoning’ the mother country for economic gain.

Socio-political variation in the intensity and manifestation of stigma can also be seen in reporting from Iraq. Iraq was one of the first countries after China to experience a significant epidemic, and given its history of political and economic instability, was unprepared (Jadoo et al., 2020). Intense stigmatization of sufferers occurred. Central government struggled to enact national policies even though willingness to comply with social distancing was relatively high, at least in educated urban populations (Jadoo et al., 2020). One particular driver of stigmatization concerned traditional death practices which are conceptualized as extremely private and family-based (Rubin, 2020). COVID-19 prevention, which requires quarantine both before and after death, was antithetical to this. Other drivers of stigma include religious beliefs concerning disease as punishment and a deep distrust of health-care providers, perceived as agents of government and feared due to high hospital mortality rates (Rubin, 2020). This tension between prevention protocols and traditional death practices has been observed in prior epidemics, such as in West Africa in relation to Ebola (Manguvo and Mafuvadze, 2015). The experience of being COVID-19 positive in the UK in mid-2020 and in Iraq during the same time period was thus very different; openness about one’s ‘Covid status’ being relatively common in the former and almost unheard of in the latter. Adapting preventive practices to create cultural acceptability is likely to lead to greater uptake (Manguvo and Mafuvadze, 2015). That said, commonalities (e.g. the prevalence of conspiracy theories concerning government) can also be observed globally. Stigma is thus both local and global at the same time, and mutates globally and locally as a consequence.

## Stigma strength

The case of Iraqi COVID-19 stigma also speaks to the final dimension of mutation I consider: the strengthening and weakening of stigma over time. ‘Stigma strength’ as a term does not denote anything about the power of any given stigma experience to affect an individual; multiple small micro-aggressions over prolonged periods can be very damaging (Sue, 2010). Rather, I use the term ‘stigma strength’ to refer to wider macro-level waves of stigma which increase and decrease over time. So far, I have focused on stigma intensifying; for example, the immediate resurgence of anti-Chinese/Asian stigma in the first COVID-19 wave. Importantly, I argue that pandemic stigma can amplify at particular cultural moments, but also weaken, producing less virulent strains, either deliberately, through anti-stigma public health interventions, or through broader cultural processes. This strengthening and weakening are also driven, particularly for COVID-19, by social media. The ‘infodemic’ offers almost limitless informational content on COVID-19 from social media platforms (e.g. Weibo, Twitter, and Facebook), most unverified by public health sources, stemming from influencers or celebrities as well as political organizations (hidden and visible; Cinelli et al., 2020). Social media can quickly amplify stigma. Tweets concerning the ‘Chinese Virus’ increased by 650% by March 2020 (Darling-Hammond et al., 2020). Equally, counter-stigmatizing Tweets can circulate globally within minutes.

### Public health interventions to mitigate stigma

Turning first to public health intervention, COVID-19 has specific features, namely its contagiousness and dangerousness to vulnerable groups, which make traditional anti-stigma interventions such as community engagement and participatory design more difficult (Logie and Turan, 2020). Roelen et al. (2020), while calling for the participation of marginalized groups in COVID-19 responses in low and middle-income countries, also acknowledge that participation itself can risk stigma. Universalism, rather than highlighting specific ‘at risk’ groups, can be effective at reducing stigma. Evidence of universalism was seen in the UK government’s message in early 2020: ‘Anyone can catch it. Anyone can spread it’. Universalist language is desirable because it both protects vulnerable groups without labelling them, and avoids stigmatizing messages based on fear or disgust, such as early public health campaigns for HIV/AIDS (Herek, 1999). However, universal messages can come to contradict other public knowledge, such as the strong social patterning of COVID-19. Public health messaging can also risk perpetuating stigma rather than countering it. A proposed NHS campaign to get ‘national treasures’ to publicly have vaccines was filled with White celebrities; a subsequent (privately funded) health campaign was produced by British Asian celebrities, given that group’s lower vaccination rates (https://www.bbc.co.uk/news/entertainment-arts-56101990, accessed 13 March 2021). Offering a processual account of pandemic stigma therefore identifies a key role for public health; to engage and disrupt stigma processes. However, given the history of public health interventions which have inadvertently or deliberately used stigmatizing or divisive tactics, this engagement with stigma processes has to be self-reflective (e.g. actively auditing the likely effect on social justice/stigma of any given intervention; Goldberg, 2017).

### Stigma weakening through de- and counter-stigmatization

Turning now to consider wider societal trends, one highly distinctive feature of the COVID-19 pandemic has been the sheer quantity of celebrities, politicians, and sports stars declaring their COVID status (Mututwa and Matsilele, 2020). In politics, this has included (the then) President Trump, EU Chief Negotiator Michael Barnier and in the UK Prime Minister Boris Johnson and Prince Charles. In the celebrity world, Tom Hank’s disclosure was followed by actor Idris Elba and countless others. In China, the actress Tang Yifei declared three of her relatives to have the virus. Given the intense taboo of declaring one’s positive status in previous epidemics, such as Ebola/HIV, this is perhaps surprising. What is distinct about COVID-19 that allows individuals, at least in most Western countries, not to fear disclosure, at the same time that COVID-19 hostility has been gathering pace?

COVID-19 is not unique in linking viral status and celebrity. Most famously, Magic Johnson, an African American basketball superstar, came out as HIV positive in 1991. His statement shifted perceptions away from HIV/AIDS as a ‘gay disease’ (Kalichman and Hunter, 1992). More recently, in the immediate aftermath of actor Charlie Sheen coming out as HIV positive, online searches for HIV/AIDS increased measurably; the ‘Charlie Sheen effect’ (Ayers et al., 2016). However, there are significant differences between the cultural history of HIV/AIDS stigma and COVID-19. The first is the delay. Magic Johnson came out as HIV positive 10 or more years into the epidemic; Charlie Sheen in 2016. COVID-19 status was openly declared within weeks. Second, as a primarily sexually transmitted disease, HIV/AIDS remains highly stigmatized. Not many celebrities have followed Magic Johnson and Charlie Sheen. Practically, the intrusive nature of the COVID-19 ‘infodemic’ has meant hiding one’s status, when in the public eye, is more difficult. Declaring oneself ‘coronapositive’ can be read both as a culturally affirmative act and a damage limitation exercise. Importantly, in socio-political terms, the declarations of openness about COVID have come from those with a voice; primarily high-status individuals with influence in the West. Even if high-status individuals possess some of the features of derogated identity, this does not lead necessarily to their devaluation (Link and Phelan, 2001).

The impact of *who* was the earliest public COVID-19 sufferer may also be important. Tom Hanks (and his wife Rita Wilson) was the ideal poster-boy for de-stigmatizing COVID-19, a wholesome successful White actor, an Oscar winner and much admired for his portrayal as a gay man with HIV/AIDS in the 1990s. Data collected within 24 hours after his disclosure showed for many, it put a (reassuring) face to a scary and unknown virus (Myrick and Willoughby, 2021). Furthermore, his immediate disclosure modelled what healthy non-stigmatized behaviour would look like in relation to knowing one’s status. Subsequently, celebrities, footballers, and politicians have disclosed their coronastatus, often posting at length, although others have hidden it (e.g. UK’s Prince William). Indeed, the ability of privileged groups to access tests early in the pandemic was the subject of backlash (Li and Shakib, 2021). Subsequently, many celebrities have posted vaccine related content. The country singer and philanthropist Dolly Parton rewrote her famous song ‘Jolene’ into ‘Vaccine’ and was videoed having her injection. From a public health perspective, the idea of harnessing social media and celebrity or influencers is highly tempting. However, public health has perhaps under-estimated the extent to which it controls celebrity content; examples of both ‘on’ and ‘off’ messaging (e.g. perpetuating conspiracy theories) are rife (Wong et al., 2020). However, what I term the ‘Hanx Effect’ demonstrates the efficacy of early high-profile anti-stigma messaging by high status individuals, and its potential to create new positive social norms.

However, celebrity behaviours do not always engender positive new norms. Celebrities perceived to be ‘tone deaf’ to the cultural atmosphere of a pandemic, such as Kim Kardashian’s private island 2020 birthday or Ellen De Generes’ joke about living in gay isolation being similar to prison, are shamed. Being perceived as good is not always about what one actually does, but what we display (Finch, 2007). Displays of care, caution, and humility, exemplified by Tom Hanks’ tweet are deemed good; displays of ostentatiousness, greed, or rule-bending (e.g. flying to Dubai for ‘work’) are deemed immoral. We can conclude, therefore, that stigma strength is an important dimension of stigma mutation. It is also highly complex; amplifying and weakening forces may be in operation simultaneously, in the media as well as elsewhere, creating further (and not always predictable) stigma mutation over time.

## Conclusion: future mutation

Stigma is the dark social shadow of biological disease. This article contributes to the sociology of stigma by offering a novel process-oriented articulation of stigma emergence and change, using the metaphor of ‘mutation’. My interest in offering this articulation has been to flesh out the ‘plagues of fear, panic, suspicion and stigma’ that Strong identified; prompted by the suddenness of COVID-19. Using the language of ‘stigma lineage’, ‘stigma variation’, and ‘stigma strength’ has allowed me to draw attention to both the continuity and change of stigma processes, so that my account of COVID-19 stigma has somewhat of a story-like quality. It tells of initial intense and reactive stigma, contextualized in particular places at particular times towards already stigmatized racial/ethnic groups, often driven by fear but also political agendas, with a multiplying of stigma possibilities or ‘variants’ as the pandemic moves on.

The story of COVID-19 stigma emergence is also a complicated one. Traditionally valued groups such as health-care workers have experienced hostility. Not all vulnerable groups, in vulnerable situations, have been highly stigmatized. Actively chosen anti-stigma language by public health, coupled with the modelling of positive social norms of disclosure, have (possibly) weakened the hold of pandemic stigma in some localities. Where does this story go next? Will inter-generational stigma be exacerbated, as young and old both move forward to claim limited resources? Will vaccine stigma emerge against those who cannot or will not be vaccinated? Perhaps the story (or stories, as they are multiply enacted) will change in unpredictable ways; it has been argued the exposure of billions of people to extreme life stress may lead to greater openness and de-stigmatization about mental health (Venkatesh and Edirappuli, 2020). Using the language of ‘mutation’ allows us to track and unpack the story of COVID-19 stigma mutation as it occurs in both global and local contexts.

There is considerable utility for the metaphor of stigma mutation beyond the COVID-19 pandemic context. In particular, identifying the three orienting concepts of lineage, variation, and strength, gives us a vocabulary for articulating how stigma exhibits continuity and change over time, as it mutates through events, interventions, and longer term cultural shifts; particularly in relation to other stigma within given socio-political locations. Crucially, stigma mutation offers an opportunity not just for process articulation, but process engagement. As we better understand the processes through which stigma mutates, this opens up the opportunity to disrupt stigma, remaking it in less deadly forms, or even preventing its emergence altogether.
